# A novel Cytochrome P450 26A1 expressing NK cell subset at the mouse maternal‐foetal interface

**DOI:** 10.1111/jcmm.16285

**Published:** 2021-01-12

**Authors:** Dan‐Ping Wei, Dan‐Dan Li, Ai‐Qin Gu, Wen‐Heng Ji, Ying Yang, Jing‐Pian Peng

**Affiliations:** ^1^ State Key Laboratory of Stem Cell and Reproductive Biology, Institute of Zoology Chinese Academy of Sciences Beijing China; ^2^ University of Chinese Academy of Sciences Beijing China

**Keywords:** Cyp26a1^+^NK cell, early pregnancy, maternal‐foetal interface

## Abstract

Cyp26a1 had important roles in mouse embryo implantation and was highly expressed in some of NK cells at the human maternal‐foetal interface in early pregnancy. However, the regulatory effect of Cyp26a1 on NK cells remains poorly understood. Through qPCR and flow cytometric assays, we found that Cyp26a1 was expressed by mouse uterine NK cells but not spleen NK cells during the peri‐implantation period and there was a group of NK cells that highly expressed Cyp26a1, that is Cyp26a1^+^NK cell subset. single cell‐population transcriptome sequencing on Cyp26a1^+^NK and Cyp26a1^−^NK cell subsets was performed. We found that there were 3957 differentially expressed genes in the Cyp26a1^+^NK cell subset with a cut‐off of fold change ≥2 and FDR < 0.01, 2509 genes were up‐regulated and 1448 genes were down‐regulated in Cyp26a1^+^NK cell subset. Moreover, cytokine‐cytokine receptor interaction signalling pathway and natural killer cell–mediated cytotoxicity signalling pathway were enriched according to KEGG pathway enrichment analysis. We further found that the expression of Gzma and Klrg1 was significantly increased and Fcgr4 was significantly decreased when inhibiting Cyp26a1. Our experimental results show that there is a novel NK cell subset of Cyp26a1^+^NK cells in mouse uterus and Cyp26a1 can regulate the gene expression of Gzma, Klrg1 and Fcgr4 in the Cyp26a1^+^NK cells.

## 1 INTRODUCTION

Natural killer (NK) cell is a type of large granular cytotoxic innate lymphoid cell that originate in the bone marrow and had no expression of T‐ or B‐cell receptors.^1^, ^2^ NK cells are widely distributed in the human body, and there is a high percentage of NK cells in the uterus.^3^ During early pregnancy, the invading trophoblast and maternal decidua form the maternal‐foetal interface, a large number of immune cells were recruited to the decidua, playing an important regulation on blastocyst implantation and placentation.^4^ Among these infiltrating immune cells, NK cells accounted for approximately 70% of the decidual leucocytes.^4^, ^5^ Decidual NK (dNK) cells played an important role in pregnancy maintenance, such as participating in decidual vascular remodelling and trophoblast invasion.^6^

Cytochrome P450 26a1 (Cyp26a1) is an oxidative metabolic enzyme, which could metabolically inactivate retinoic acid by converting it into more polar hydroxylated and oxidized derivatives.^7^, ^8^, ^9^ Mice of Cyp26a1‐null mutants die in the late second trimester of pregnancy with many major morphogenesis defects.^10^ Previous research in our laboratory showed that Cyp26a1 was specifically and highly expressed at the maternal‐foetal interface in mouse early pregnancy; meanwhile, the pregnancy rate and the number of implantation sites were significantly decreased in Cyp26a1 plasmid‐immunized or Cyp26a1‐specific antisense oligos treated mice.^11^ Further study of our laboratory found that the proportion of NK cells was significantly changed in the uterus in early pregnancy in the above two mouse models.^12^ There was a report showing that Cyp26a1 was highly expressed in dNK1 at the human first‐trimester maternal‐foetal interface.^13^ Moreover, Cyp26a1 was highly expressed in dNK cells (decidua NK cells) and lowly expressed in pNK cells (peripheral blood NK cells) of human.^14^ However, it has not been reported whether the specific expression of Cyp26a1 in NK cells played a special regulatory role.

In this study, our research discovered that there was a Cyp26a1^+^NK cell subset at the mouse maternal‐foetal interface in early pregnancy and we performed preliminary research on this NK subset.

## 2 MATERIALS AND METHODS

### 2.1 Mice

BALB/c mice of 6‐8 weeks were purchased from Beijing Vital River Laboratory Animal Technology Co, Ltd, and 8‐12 weeks during the experiment. All animal manipulation procedures were approved by the Institutional Animals Care and Use Committee of the Institute of Zoology, Chinese Academy of Sciences. In the afternoon of the previous day, male mice and female mice were caged in a ratio of 1:1, and the vaginal plugs of female mice were detected in the next morning. The presence of vaginal plugs was recorded as the first day of pregnancy (gd1).

### 2.2 RNA extraction and quantitative reverse transcription polymerase chain reaction (qPCR)

Total RNA of cells or tissues was extracted according to the TRIzol (15596, ambion) reagent instructions. Then, cDNA was synthesized using the M‐MLV reverse transcriptase system (M170, Promega). qPCR experiment performed on the Roche LightCycler 480 instrument, using UltraSYBR Mixture (CW0957, CWBIO). The 2^−ΔΔ^ *^C^* ^t^ method was used to analyse the changes of gene expression in the experimental data. The target gene of qPCR primers was shown in Table S1.

### 2.3 Western blotting

Grind mouse uterine tissue into powder in liquid nitrogen and then added to RIPA Lysis Buffer (C1053, APPLYGEN), simultaneously adding protease inhibitor cocktail (HX1863, huaxingbio). The total protein was extracted according to the instructions of the RIPA Lysis Buffer. The protein concentration was determined by BCA protein quantification kit (23227, Pierce) according to the instructions; then, SDS‐PAGE was used to separate protein. The protein on the gel was transferred to the NC membrane. After blocking with 5% skimmed milk, incubating with primary antibody at 4°C overnight and secondary antibody at room temperature 1 hour, and then adding West Pico PLUS chemiluminescent substrate (34579, Thermo Fisher), immunoreactive protein bands were detected and imaging by Genegnome instrument. The primary antibodies used were anti‐CYP26A1 (PA5‐24602, Invitrogen, 1:1000) and anti‐GAPDH (2118, CST, 1:1000). The secondary antibody used was Goat anti‐Rabbit IgG (H + L) HRP (31460, Thermo Fisher, 1:10 000).

### 2.4 Preparation of mouse uterine single cell suspension

Referring to the method in the previous article with minor modifications,^15^ the main steps were as follows. After the mice were sacrificed, the uterine tissues were cut out, washed once with 1xPBS, shredded in digestion mixture and digested at 37°C in a shaker for 30 minutes. The digestion mixture was prepared with RPMI Media 1640 and contained 1 mg/mL collagenase IV (C5138, Sigma‐Aldrich), 0.3 mg/mL hyaluronidase (H3506, Sigma‐Aldrich) and 8% FBS. After digestion, digestive fluid was removed by centrifuge, 1×PBS was added into tissue and cell mixture and incubate at 37°C in a shaker for 15 minutes, and then filter with a 400 mesh sieve to obtain a single cell suspension of uterus.

### 2.5 Preparation of mouse spleen single cell suspension

Refer to the previously mentioned with small modifications.^16^ The main method was as follows. A fresh spleen of mouse was put into 1×PBS and shredded to small pieces. Then through filter with a 100 mesh sieve. Added 3 volumes red blood cell lysate (40401ES60, YEASEN) to cell suspension and centrifuge for 5min, then discard the supernatant. Add 1×PBS to resuspend the cells and filter with a 400 mesh sieve. Spleen single cell suspension was obtained after washed again with 1×PBS.

### 2.6 Flow cytometric assays

Fc antibody (14‐0161, eBioscience) was added to the single cell suspension to prevent non‐specific antibody binding at 4°C for 10 minutes; then, single‐cell suspension was incubated with antibody at 4°C for 30 minutes. After incubation, cells were washed with 1×PBS and resuspended in 1×PBS，then they were analysed on the instrument BD AriaFusion or BD FACSAria IIIu. Antibodies used included: anti‐CD3‐PE (12‐0031, eBioscience), anti‐CD45‐PerCP‐Cy5.5 (45‐0451, eBioscience), anti‐CD122‐APC (17‐1222, eBioscience), anti‐CYP26A1 (PA5‐24602, Invitrogen, 1:25) and it's corresponding fluorescent secondary antibody anti‐Rabbit Alexa Fluor 488 (A‐21206, Thermo Fisher, 1:500). We define CD45^+^CD3^‐^CD122^+^ cells as NK cells, CD45 was the leucocyte common antigen, CD122 was used to label NK cells, and CD3 was used to exclude T cells.^17^, ^18^ Data of flow cytometric assays were analysed with FlowJo.

### 2.7 Immunocytofluorescence

CD45^+^CD3^‐^CD122^+^ NK cells were purified by flow cytometric sorting according to method of 2.6 flow cytometric assays; antibodies used included: anti‐CD3‐PE (12‐0031, eBioscience), anti‐CD45‐PerCP‐Cy5.5 (45‐0451, eBioscience) and anti‐CD122‐APC (17‐1222, eBioscience). Then, those viable and unfixed NK cells were incubated with anti‐CYP26A1 (PA5‐24602, Invitrogen, 1:25) at 4°C for 1 hour and then incubated with anti‐Rabbit Alexa Fluor 488 (A‐21206, Thermo Fisher, 1:500) at the same condition. Finally, the cells suspension were dropped onto the glass slide and covered with a coverslip. Zeiss LSM 780 instrument was used to capture images, and ZEN software was utilizated to process the data. CD45^+^CD3^‐^CD122^+^Cyp26a1^+^NK cells and CD45^+^CD3^‐^CD122^+^Cyp26a1^−^NK cells were obtained from the uterus of pregnant mouse on gd5 by flow cytometric sorting. The two sorted NK cell subsets were directly capture images using Zeiss LSM 780 instrument and analysed in the same way as above.

### 2.8 Immunohistofluorescence

The paraffin sections (4 μm) of mouse (BALB/c strain) uterus on gd6 were mounted on 3‐Aminopropyl‐Triethoxysilane‐coated slides. The sections were blocked in 5% donkey serum at room temperature for 1 hour after deparaffinized, rehydrated and citric acid antigen retrieval. Then, the sections were incubated with rabbit anti‐Cyp26a1 antibody (ab151968, abcam, 1:100) and goat anti‐Nkp46 antibody (AF2225, R&D Systems, 1:50) overnight. After washing thoroughly in PBS, the sections were incubated with donkey anti‐rabbit IgG Alexa Fluor 488 conjugated (A‐21206, Thermo Fisher, 1:250) and donkey anti‐goat IgG Alexa Fluor 647 conjugated (A‐21447, Thermo Fisher,1:250) at room temperature for 1 hour. Finally, the sections were mounted by Aqueous Mounting Medium with DAPI (sc‐24941, Santa Cruz). The images were capture by Zeiss LSM 880 instrument.

### 2.9 Immunohistochemistry

The operation method of paraffin section was the same as 2.9 Immunohistofluorescence, until the sections were incubated with rabbit anti‐Cyp26a1 antibody (ab151968, abcam, 1:100) overnight. The frozen sections (5 μm) of mouse (BALB/c strain) uterus of gd6 were mounted on 3‐Aminopropyl‐Triethoxysilane‐coated slides. The frozen sections were treated with 0.3% Triton X‐100 for 10 minutes and blocked in 5% BSA at room temperature for 1 hour and after the embedding agent was washed. Then, the frozen sections were incubated with goat anti‐Nkp46 antibody (AF2225, R&D Systems, 1:50) overnight. The next day, the 3% H_2_O_2_ was used to treat both of the paraffin sections and frozen sections for 15 minutes to inhibit endogenous peroxidase activity. After washing thoroughly in PBS, all the sections were incubated with HRP‐conjugated secondary antibody (1:200) at room temperature for 1 hour. The colour was developed with diaminobenzidine tetrahydrochloride (DAB) kit (ZLI‐9017, ZSGB‐BIO). Images were gotten by the Nikon ECLIPSE Ni‐U microscope.

### 2.10 Single cell‐population transcriptome sequencing

Purified CD45^+^CD3^−^CD122^+^Cyp26a1^+^NK cells and CD45^+^CD3^−^CD122^+^Cyp26a1^−^NK cells were collected from the uterus of pregnant mouse on gd5 by flow cytometric sorting using BD FACSAria IIIu. Total RNA of the two NK cell subsets were extracted according to the TRIzol (15596, ambion) reagent instructions. The purity of RNA was tested using NanoDrop 2000 micro‐spectrophotometer. The concentration and integrity of RNA were tested using Agilent 2100 Bioanalyzer and Agilent RNA 6000 Pico Kit. Transcriptome library was constructed following SMART‐seq2 protocol, and transcriptome sequencing data were obtained based on the illumina NovaSeq 6000 sequencing technology platform. Sequencing reads were aligned to the mouse genomes (mm10) by using the HISAT2 (tophat) with the default parameters. Total read counts for each protein‐coding gene were extracted using HTSeq (version 0.6.0) and then loaded into R package DESeq2 to calculate differentially expressed genes with cut‐off of fold change ≥ 2 and FDR < 0.01. The GO and KEGG analyses of differentially expressed genes were performed using R package clusterProfiler.

### 2.11 Cell culture and drug treatment

CD45^+^ cells were isolated from the uterus of pregnant mouse on gd5 by flow cytometric sorting and cultured in RPMI‐1640 medium (Gibco) supplemented with 10% foetal bovine serum (FBS, Gibco) and 1% penicillin/streptomycin. Cells were maintained at 37ºC with 5% CO2 in a humidified chamber. Cyp26a1 inhibitor R115866 (SML2092, sigma) were dissolved in dimethyl sulfoxide (DMSO) to 10 mmol/L, then divided into small portions to store at −20°C. Cells were incubated with R115866 (10 μmol/L) or Cyp26a1 antibody (PA5‐24602, Invitrogen, 80 μg/mL) for 12 hours before collected for qPCR detection. The control groups were treated with 0.1% DMSO or normal IgG (A01008, Genscript, 80 μg/mL) in equal time.

### 2.12 Statistical analysis

The data were analysed by unpaired *t* test analysis using GraphPad Prism version 7. The data value was presented as mean ± SEM, *represents *P* < .05, **represents *P* < .01, ***represents *P* < .001 and **** represents *P* < .0001.

## 3 RESULTS

### 3.1 New Cyp26a1^+^NK cell subset

#### 3.1.1 Expression pattern of Cyp26a1 in mouse uterine tissue during the peri‐implantation period

**FIGURE 1:**
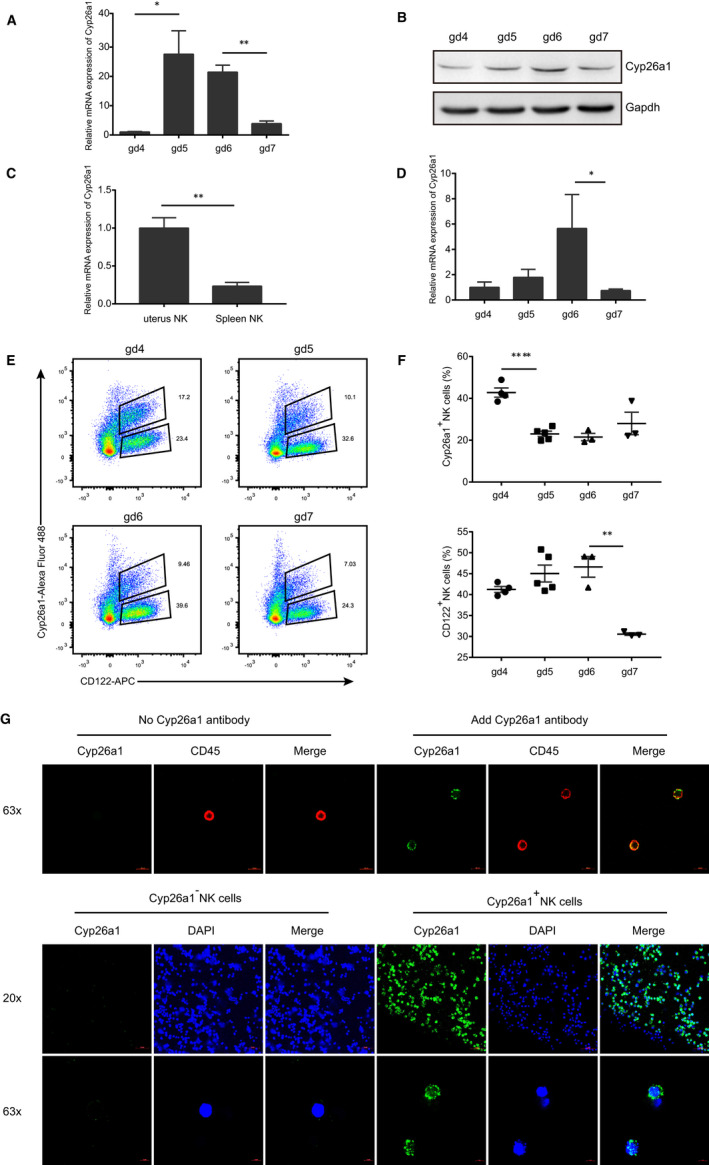
Expression of Cyp26a1 in mouse uterine tissue and uterine NK cells during the peri‐implantation period. (A) qPCR to detect the expression of Cyp26a1 on gd4‐gd7 uterine tissue. (B) Western blotting to detect the expression of Cyp26a1 on gd4‐gd7 uterine tissue. (C) qPCR to detect the expression of Cyp26a1 in CD45^+^CD3^−^CD122^+^NK cells isolated from the uterus and spleen on gd5. (D) qPCR to detect the expression of Cyp26a1 in CD45^+^CD3^−^CD122^+^NK cells isolated from the uterus on gd4‐gd7. (E) Representative graph of flow cytometric assays to detect CD45^+^CD3^−^CD122^+^Cyp26a1^+^NK cells in the uterus on gd4‐gd7, the marker molecules were as follows: CD3‐PE, CD45‐PerCP‐Cy5.5, CD122‐APC and Cyp26a1‐Alexa Fluor 488, The cells under gate CD45^+^CD3^‐^shown in the figure. (F) Percentages of Cyp26a1^+^NK cells in gated CD45^+^CD3^−^CD122^+^NK and CD122^+^ NK cells in gated CD45^+^CD3^−^cells in the uterus on gd4‐gd7. (G) Upper picture: immunofluorescence detection of Cyp26a1 expression in CD45^+^CD3^−^CD122^+^NK cells isolated from the uterus on gd5 (63 × objective); lower picture: confocal photography of Cyp26a1^+^NK cells and Cyp26a1^‐^NK cells isolated from the uterus on gd5 (20 × and 63 × objective). Data were means ± SEM from three biological replicates, *represents *P* < .05, **represents *P* < .01 (two‐tailed unpaired Student's *t* test) (A and C). Data were representative of two (A, B and C) or three (E, F and G) independent experiments

qPCR and Western blotting experiments were conducted to detect Cyp26a1 expression in mouse uterine tissue during the peri‐implantation period. The results showed that Cyp26a1 was specifically and dynamically expressed in the peri‐implantation in mouse uterine tissue, which was lowly expressed on gd4 before embryo implantation, highly expressed on gd5 and gd6 after embryo implantation, and then down‐regulated after gd6 (Figure 1A‐B).

#### 3.1.2 Expression pattern of Cyp26a1 in mouse uterine NK cells

The results of qPCR on CD45^+^CD122^+^CD3^−^NK cells isolated from the uterus and spleen on gd5 and qPCR on CD45^+^CD122^+^CD3^‐^NK cells isolated from the uterus on gd4‐gd7 showed that Cyp26a1 was highly expressed in NK cells in the uterus and lowly expressed in NK cells in the spleen (Figure 1C), in addition, the expression trend of Cyp26a1 in NK cells in the uterus appeared a dynamic change during the peri‐implantation period, which is similar to Cyp26a1 expression pattern in uterine tissue (Figure 1D).

#### 3.1.3 Grouping and verification of NK cells expressing Cyp26a1

In order to explore whether NK cells expressing Cyp26a1 could form a subset, we used anti‐Cyp26a1 antibody to label uterine NK cells, and combined with Alexa Fluor 488 fluorescent secondary antibody to perform flow cytometric assays, what surprised us was that NK cells in the uterus on gd4‐gd7 can be divided into two cell subsets of CD45^+^CD122^+^CD3^−^Cyp26a1^+^NK (Cyp26a1^+^NK) and CD45^+^CD122^+^CD3^−^Cyp26a1^−^NK (Cyp26a1^−^NK) (Figure 1E). The gating strategy and complete data were shown in Figure S1. The ratio of Cyp26a1^+^NK was high on gd4 and then showed a downward trend (Figure 1F). Meanwhile, we also found that dynamic change of CD45^+^CD122^+^CD3^‐^NK cells was similar the expression pattern of Cyp26a1 in uterine tissue (Figure 1F). To further confirm the expression of Cyp26a1 in uterine NK cells, immunofluorescence experiment was performed on CD45^+^CD122^+^CD3^−^NK cells isolated from the uterus on gd5 by flow cytometric sorting. The results showed that the experimental group added Cyp26a1 antibody displayed clear green signal of Cyp26a1 on the cell membrane comparing with the control group (Figure 1G). Moreover, we conducted laser confocal microscopy analysis of the Cyp26a1^+^NK cells and Cyp26a1^−^NK cells isolated from the uterus on gd5 by flow cytometric sorting. There were obvious green signals of Cyp26a1 on the Cyp26a1^+^NK cell subset, while the Cyp26a1^−^NK cell subset had no detectable green signals (Figure 1G).

### 3.2 Localization of Cyp26a1^+^NK cells

**FIGURE 2:**
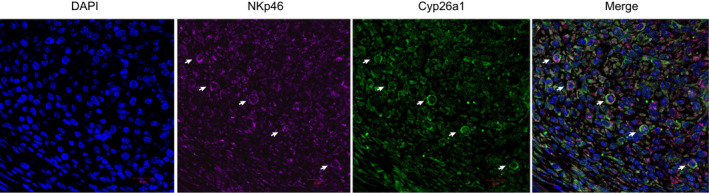
Localization of Cyp26a1^+^NK cells in the uterus on gd6. Immunohistofluorescence images of uterus sections were co‐stained for Cyp26a1 and NKp46; co‐localization of Cyp26a1 and NKp46 were shown by the white arrows, scale bar: 20 μm

In order to study the location of Cyp26a1^+^NK cells in the uterus, the immunohistochemical experiment of Cyp26a1 and NKp46 localization on mouse gd6 uterine tissue using anti‐Cyp26a1 or anti‐NKp46 antibodies was performed (Figure S2B). Meanwhile, Immunohistofluorescence experiments using anti‐Cyp26a1 and anti‐NKp46 antibodies co‐stained were also performed to detect the co‐localization of Cyp26a1 and NKp46 on mouse gd6 uterine tissue (Figure 2 and Figure S2A).

### 3.3 Single cell‐population transcriptome sequencing and analysis of Cyp26a1^+^NK and Cyp26a1^‐^NK cell subsets in uterus on gd5

**FIGURE 3:**
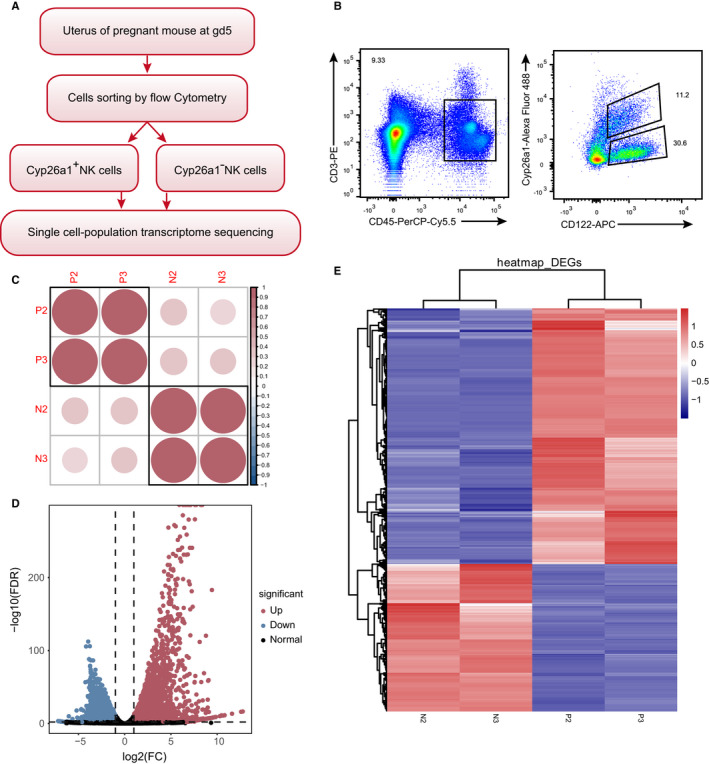
Transcriptome sequencing and differentially expressed gene analysis (A) Flow chart of transcriptome sequencing. (B). Flow cytometric sorting diagram of transcriptome sequencing samples, Cyp26a1^+^NK cells (CD45^+^CD3^‐^CD122^+^Cyp26a1^+^) and Cyp26a1^−^NK cells (CD45^+^CD3^‐^CD122^+^Cyp26a1^‐^) were isolated from the mouse uterus on gd5. (C) Sample consistency analysis graph, ‘P2 and P3’ represented Cyp26a1^+^NK cells and ‘N2 and N3’ represented Cyp26a1^−^NK cells in the C, D and E. (D) Volcano plot map. (E) Hierarchical clustering demonstrating a distinguishable gene expression pattern between Cyp26a1^+^NK cells and Cyp26a1^−^NK cells

To investigate the function of Cyp26a1^+^NK cells, single cell‐population transcriptome sequencing was performed on Cyp26a1^+^NK and Cyp26a1^−^NK cell subsets isolated from the uterus on gd5 by flow cytometric sorting (Figure 3A‐B). Figure 3C showed that the repeatability between samples was well. Volcano plot and hierarchical clustering demonstrated a distinguishable gene expression pattern between Cyp26a1^+^NK cell Cyp26a1^−^NK cell subsets (Figure 3D‐E). Compared with the Cyp26a1^−^NK cell subset, there were 3957 differentially expressed genes in the Cyp26a1^+^NK cell subset with cut‐off of fold change ≥ 2 and FDR < 0.01. 2509 genes were up‐regulated in Cyp26a1^+^NK cell subset, and 1448 genes were down‐regulated (Table S2).

**FIGURE 4:**
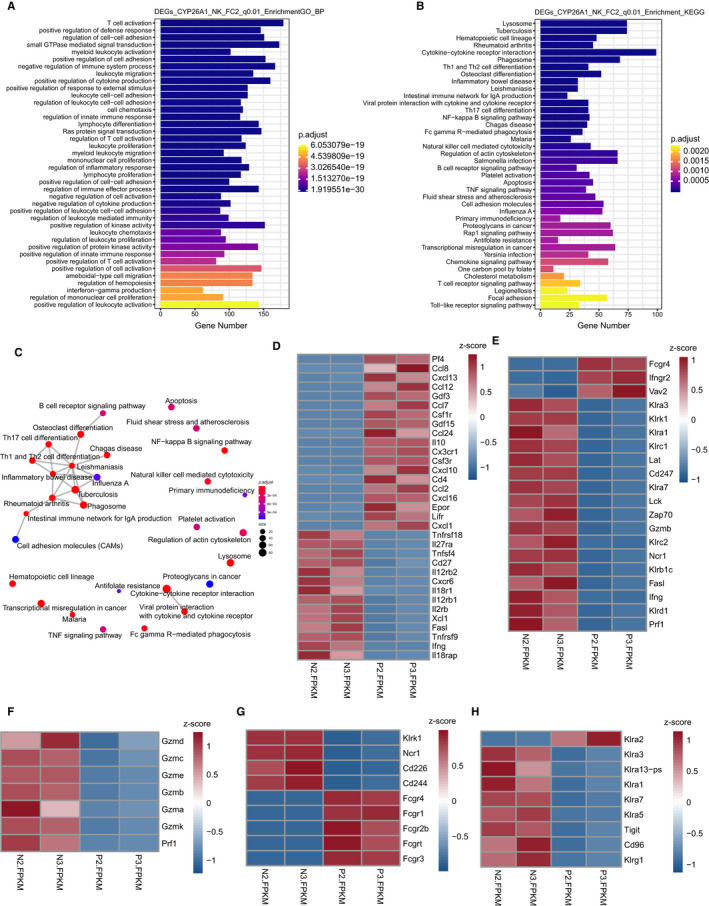
GO and KEGG analyses. (A) GO analysis of gene enriched in biological processes. (B) KEGG analysis of the significantly enriched pathways. (C) Gene overlap relationship between enriched pathways. Enrichment factor is the ratio of the number of genes enriched in a signal pathway to the number of all genes annotated in this pathway; p.adjust is corrected p‐value; the colour and size of the dots represent the p.adjust and the number of genes enriched in indicated pathways, respectively. (D) Z‐scores of part of gene enriched in cytokine‐cytokine receptor interaction. (E) Z‐scores of part of gene enriched in natural killer cell–mediated cytotoxicity, ‘P2 and P3’ represented Cyp26a1^+^NK cells and ‘N2 and N3’ represented Cyp26a1^‐^NK cells in the D‐H. (F) Z‐scores of expression of granzyme and perforin. (G‐H) Z‐scores of activating receptor and inhibitory receptor

Furthermore, we performed GO and KEGG analyses. According to the results of GO analysis, we found that the enriched biological processes include T cell activation, myeloid leucocyte activation and negative regulation of immune system process (Figure 4A). KEGG analysis showed that cytokine‐cytokine receptor interaction, Fc gamma R‐mediated phagocytosis and natural killer cell–mediated cytotoxicity were enriched (Figure 4B‐C). Pf4 and Ccl12 were highly expressed in Cyp26a1^+^NK cell subset, and Tnfrsf9 and Fasl were lowly expressed in Cyp26a1^+^NK cell subset (Figure 4D). Fcgr4 and Tnf were highly expressed in Cyp26a1^+^NK cell subset, and Gzmb, Fasl and Prf1 were lowly expressed in Cyp26a1^+^NK cell subset (Figure 4E). Granzyme and perforin were lowly expressed in Cyp26a1^+^NK cells (Figure 4F). The activating receptor Fcgr4, etc, were highly expressed in Cyp26a1^+^NK cells, yet, Klrk1 (NKG2D), Ncr1 (NKp46), Cd226 (DNAM‐1) and Cd244a (2B4) were lowly expressed (Figure 4G). The inhibitory receptor Klra2 (Ly49B) was highly expressed in Cyp26a1^+^NK cells, while Cd96 and Klrg1, etc, were lowly expressed (Figure 4H). Classification the activating receptor and the inhibitory receptor were reference from previous reports.^19^

### 3.4 Verification of single cell‐population transcriptome sequencing

**FIGURE 5:**
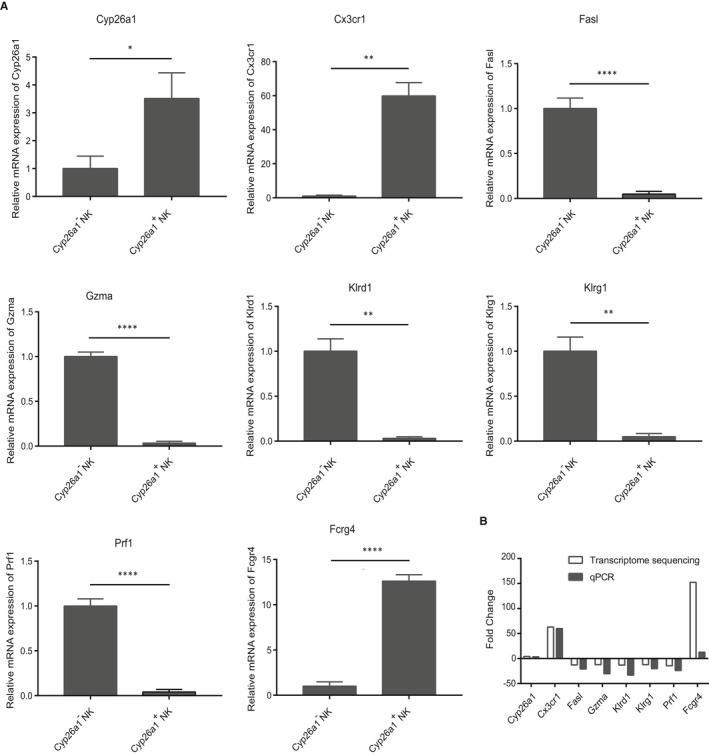
Verification of single cell‐population transcriptome sequencing. (A) qPCR to detect the expression of Cyp26a1, Cx3cr1, Fasl, Gzma, Klrd1, Klrg1, Prf1 and Fcrg4 in CD45^+^CD3^−^CD122^+^Cyp26a1^+^NK cells and CD45^+^CD3^−^CD122^+^Cyp26a1^‐^NK cells isolated from the uterus on gd5. (B) Comparison between qPCR and transcriptome sequencing results. Data were means ± SEM from five biological replicates, * represents *P* < .05, ** represents *P* < .01, *** represents *P* < .001 and **** represents *P* < .0001 (two‐tailed unpaired Student's *t* test)

Cyp26a1 and several NK cell–related genes were chosen for qPCR experiments to verify the transcriptome sequencing results in Cyp26a1^+^NK cells and Cyp26a1^−^NK cells isolated from the mouse uterus on gd5. The results of qPCR showed that Cyp26a1 and Cx3cr1 mRNA were highly expressed in the Cyp26a1^+^NK cell subset; however, FasL, Gzma, Klrd1, Klrg1 and prf1 were lowly expressed in the Cyp26a1^+^NK cell subset (Figure 5A). Furthermore, qPCR results were largely consistent with the single cell‐population transcriptome sequencing results (Figure 5B).

### 3.5 Changes of NK cell function‐related molecules after inhibiting Cyp26a1

**FIGURE 6:**
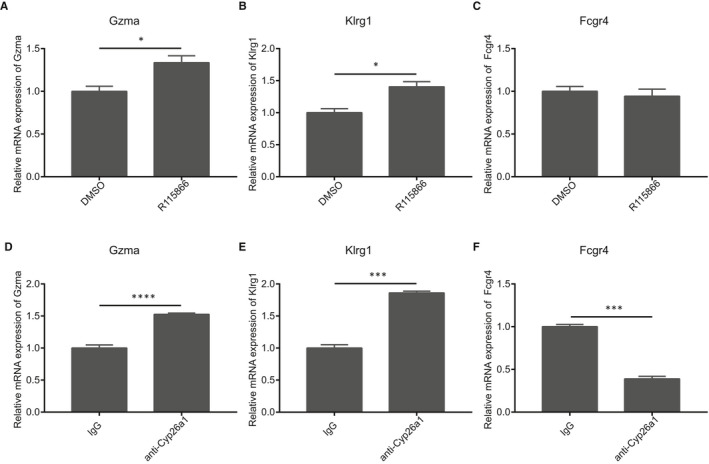
Changes of genes related to NK cells function after inhibiting Cyp26a1 (A‐F) qPCR to detect the expression of Gzma, Klrg1 and Fcgr4 in CD45^+^ lymphocytes isolated from the uterus on gd5 which were treated 12 h by 10 μmol/L Cyp26a1 inhibitor R115866 or 80 μg/mL Cyp26a1 antibody and the control group were treated with the same amount of DMSO (0.1%) or normal IgG (80 μg/mL) for equal time. Data were means ± SEM from three replicates, * represents *P* < .05, ** represents *P* < .01, *** represents *P* < .001 and **** represents *P* < .0001 (two‐tailed unpaired Student's *t* test). Data were representative of two (D) or three (A‐C, E‐F) independent experiments

To evaluate the regulatory effect of Cyp26a1 on NK cell function‐related molecules, we isolated CD45^+^lymphocytes from the uterus on gd5, and then treated with the Cyp26a1 inhibitor R115866 or Cyp26a1 antibody. Gzma and Klrg1 were up‐regulated in both Cyp26a1 inhibitors R115866‐treated and Cyp26a1 antibody‐treated group when compared with the control (DMSO or IgG) group (Figure 6A‐B, Figure 6D‐E). At the same time, we also found that after treating CD45^+^lymphocytes with the Cyp26a1 antibody, the expression of Fcgr4 was down‐regulated relative to the control group (Figure 6F). However, Cyp26a1 inhibitor R115866 treatment did not cause changes in Fcgr4 in comparison with the control group (Figure 6C).

## 4 DISCUSSION

We found that Cyp26a1 was dynamically and specifically expressed in the mouse uterine tissue during the peri‐implantation period, and the expression level increased remarkably at D5 and then gradually declined (Figure 1A‐B). These observations were consistent with previous research in our laboratory.^11^, ^20^, ^21^ We also found that Cyp26a1 was expressed in mouse uterine NK cells, but not in spleen NK cells (Figure 1C), which was consistent with the report in human NK cells.^13^, ^14^ There were a variety of NK cell subsets at the human maternal‐foetal interface, and different NK subsets had different gene expression profiles and functions.^13^, ^22^ Previous studies in our laboratory had shown that Cyp26a1 could affect pregnancy outcomes by regulating immune cells, such as regulating Th17 cells, affecting the iDC/mDC ratio and the ratio of NK cells.^12^, ^23^, ^24^ In this study, we discovered that there was a Cyp26a1^+^NK cell subset in the uterus of mouse in early pregnancy by flow cytometric assays (Figure 1E).

To study the function of Cyp26a1^+^NK cells, we conducted single cell‐population transcriptome sequencing. qPCR verification showed that single cell‐population transcriptome sequencing was reliable (Figure 5B). Cyp26a1^+^NK cells had low expression of granzyme and perforin (Figure 4, Figure 5), which indicates that Cyp26a1^+^NK cells may have limited cytotoxicity function.

Our further in vitro experiments found that Gzma and Klrg1 expression increased after treatment with Cyp26a1 inhibitor R115866 or Cyp26a1 antibody, and Fcgr4 expression decreased after treatment with Cyp26a1 antibody but not Cyp26a1 inhibitor R115866 (Figure 6). R115866 is an inhibitor which could prevent retinoic acid from being metabolized by Cyp26a1.^25^

Previous research in our laboratory showed that the number of implantation sites and Th17 levels in the implantation sites were significantly reduced when mouse was treated by anti‐cyp26a1 antibody, which implied that Cyp26a1 may play an important role in mouse embryo implantation and immune modulation.^11^, ^24^ Gzma in NK cell could induce pro‐inflammatory cytokine response and pyroptosis of target cells.^26^, ^27^ Klrg1 (Killer cell lectin‐like receptor G1) is a C‐type lectin‐like receptor which binds to cadherins (E‐, N‐ and R‐) on target cells, and inhibit NK cell cytotoxicity.^28^, ^29^ Fcgr4 expresses the FcγRIV protein, which is involved in NK cell‐mediated antibody‐dependent cellular cytotoxicity (ADCC) by binding to IgG2a and IgG2b.^30^ Our results showed that the expression of Fcgr4 was not positively correlated with gzma in Cyp26a1^+^NK cells. This may be because the cells themselves store Gzma, but it is still unclear and needs to be investigated further. We used CD45‐labelled positive cells instead of CD45^+^CD3^‐^CD122^+^ NK cells isolated from the uterus on gd5 when studying the regulation of Cyp26a1 on NK cell function. Our main consideration for doing so is summarized by the following three aspects: (a) Cells grew better and the microenvironment is closer to that of the in vivo state when a variety of cells were cultured in a pool. (b) The isolated NK cells are unstable and had abnormal gene expression when cultured alone. (c) NK cells account for a high proportion of total CD45^+^lymphocytes and the genes we tested were highly expressed in NK cells.

Figure 1A, Figure 1B and Figure 1D showed the expression of Cyp26a1 was low on gd4, and Figure 1E and Figure 1F showed that the population of Cyp26a1^+^NK cell was the high. This may be due to the following reasons: Figure 1A and Figure 1B detects the expression of Cyp26a1 in all cells of the uterus. Figure 1D detects the expression of Cyp26a1 in total NK cells, Figure 1E and Figure 1F shows the proportion of Cyp26a1^+^NK cells in CD45^+^CD3^‐^cells and total NK cells, respectively. NK cells account for a few proportion of uterine cells. The expression of Cyp26a1 in uterine tissue mainly comes from glandular epithelial and luminal epithelial cells.^11^, ^21^

There were several drawbacks in this study. The actual function of Cyp26a1^+^NK cells was unclear. Our finding that Cyp26a1 regulates the function of NK cells through Gzma, Klrg1 and Fcgr4 needs to be verified in vivo.

**FIGURE 7:**
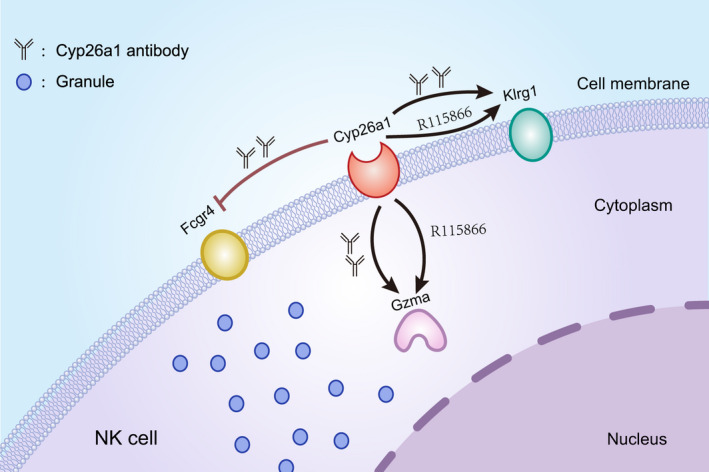
Diagram the regulation of Cyp26a1 on NK cell functional molecules. The schematic diagram of NK cells shows the regulation of Cyp26a1 on Gzma(granzyme A), Klrg1(killer cell lectin‐like receptor subfamily G, member 1) and Fcgr4(Fc receptor, IgG, low affinity IV) in NK cells. Gzma and Klrg1 were up‐regulated when inhibit Cyp26a1 by Cyp26a1 inhibitor R115866 or Cyp26a1 antibody. Blocking Cyp26a1 by Cyp26a1 antibody, Fcgr4 was down‐regulated. Black arrows represent promotional effects. The red blocking symbol represents inhibitory effects. The Y‐shaped figure represents the blocking antibody of Cyp26a1. R115866 is an inhibitor of Cyp26a1

In conclusion, our data illustrated that there was a subset of Cyp26a1^+^NK cells at the mouse maternal‐foetal interface and this group of cells has low expression of NK cell toxicity‐related genes. We speculate that Cyp26a1 has a modulating effect on NK cells during the peri‐implantation period in mouse and this effect may be exercised through Gzma, Klrg1 and Fcgr4 (Figure 7).

## CONFLICT OF INTEREST

The authors confirm that there are no conflicts of interest.

## AUTHOR CONTRIBUTION

**Dan‐Ping Wei:** Data curation (lead); Investigation (lead); Methodology (lead); Writing‐original draft (lead). **Dan‐Dan Li:** Methodology (supporting). **Ai‐Qin Gu:** Methodology (supporting). **Wen‐Heng Ji:** Methodology (supporting). **Ying Yang:** Methodology (supporting). **Jing Pian Peng:** Funding acquisition (lead); Methodology (supporting); Resources (lead).

## Supporting information

Fig S1

Fig S2

Table S1

Table S2

## Data Availability

We confirm that the data in our paper can be used.
