# Supplementary figures and images for: A novel Cytochrome P450 26A1 expressing NK cell subset at the mouse maternal‐foetal interface
Source: J Cell Mol Med. 2021 Jan 12;25(3):1771–82. doi: 10.1111/jcmm.16285 (PMC7875917; doi:10.1111/jcmm.16285)

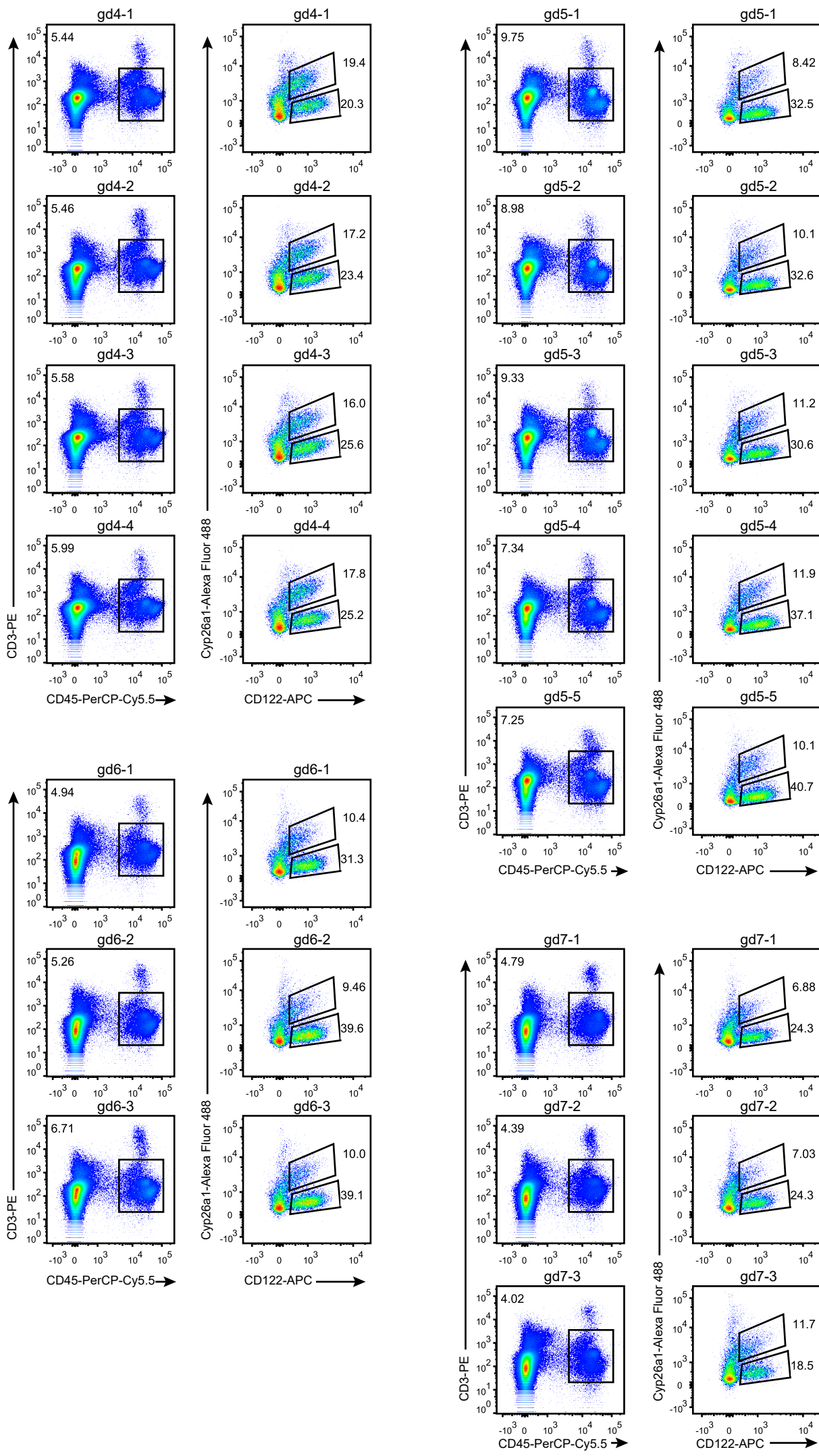

Supplement: Supplementary file 1 — Fig S1 [file JCMM-25-1771-s001.pdf]

**A**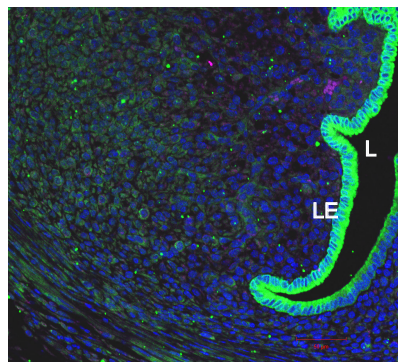**B**

Cyp26a1

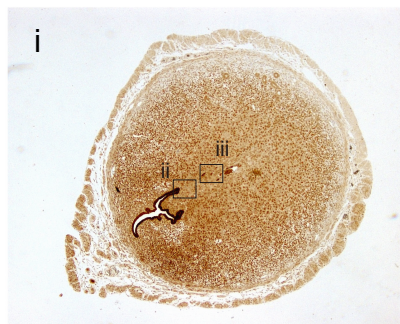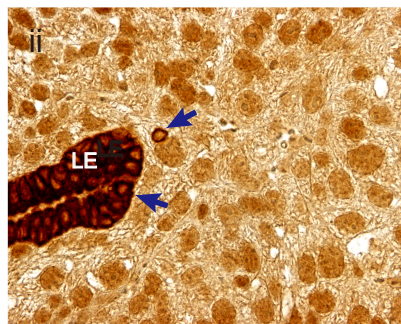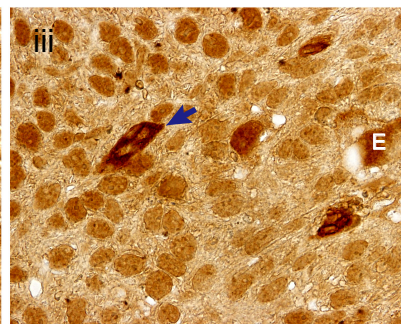

NKp46

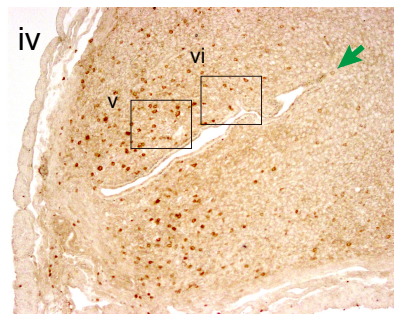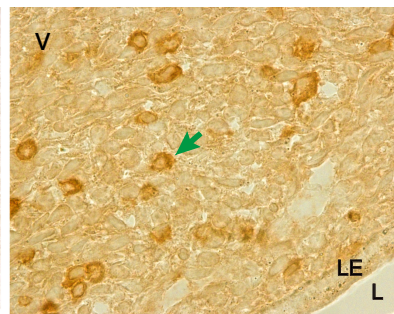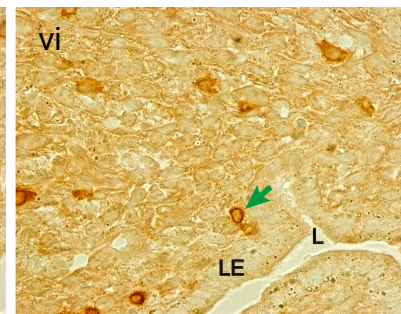

Supplement: Supplementary file 2 — Fig S2 [file JCMM-25-1771-s002.pdf]
