# Supplementary material for: A novel Cytochrome P450 26A1 expressing NK cell subset at the mouse maternal‐foetal interface
Source: J Cell Mol Med. 2021 Jan 12;25(3):1771–82. doi: 10.1111/jcmm.16285 (PMC7875917; doi:10.1111/jcmm.16285)
Supplement: Supplementary file 3 — Table S1 [file JCMM-25-1771-s003.doc]

Table S1. Mouse qPCR primer sequences.

| Genes | Oligonucleotides（5’-3’） |
| --- | --- |
| Primers for mouse Cyp26a1 | Forward：AGGCTGGATATGCAGGCACTA  Reverse：GTTGTCTTGATTGCTCTTGCAAAGT |
| Primers for mouse Cx3cr1 | Forward：TCTGCGTGAGACTGGGTGAG  Reverse：CGTGAAGACGAGGGCGTAGA |
| Primers for mouse FasL | Forward：CCAACCCCAGTACACCCTCT  Reverse：GGCCACCTTTCTTATACTTCACTCC |
| Primers for mouse Gzma | Forward：TGCTGCCCACTGTAACGTG  Reverse：GGTAGGTGAAGGATAGCCACAT |
| Primers for mouse Klrd1 | Forward：TCTAGGATCACTCGGTGGAGA  Reverse：CACTTGTCCAGGCAAACACAG |
| Primers for mouse Klrg1 | Forward：TACACCGGCCCCATCTTTCC  Reverse：AGTGGCTACCATTCCTCGTCC |
| Primers for mouse Prf1 | Forward：TGAACCCTAGGCCAGAGGCA  Reverse：TAAAGTTGCGGGGGAGGGCT |
| Primers for mouse Fcgr4 | Forward：ATGTGGCAGCTACTACTACCA  Reverse：ACCCACTTGGGGTCTAGGTTC |
| Primers for mouse Gapdh | Forward：AGGTCGGTGTGAACGGATTTG  Reverse：TGTAGACCATGTAGTTGAGGTCA |
| Primers for mouse Actb | Forward：AAGGCCAACCGTGAAAAGAT  Reverse：GTGGTACGACCAGAGGCATAC |
